# Factors Associated with Insomnia among Elderly Patients Attending a Geriatric Centre in Nigeria

**DOI:** 10.1155/2014/780535

**Published:** 2014-12-22

**Authors:** Adetola M. Ogunbode, Lawrence A. Adebusoye, Olufemi O. Olowookere, Mayowa Owolabi, Adesola Ogunniyi

**Affiliations:** ^1^Department of Family Medicine, University College Hospital, PMB 5116 Agodi, Ibadan 200221, Nigeria; ^2^Chief Tony Anenih Geriatric Centre (CTAGC), University College Hospital, Ibadan, Nigeria; ^3^Department of Medicine, College of Medicine, University of Ibadan, Nigeria

## Abstract

*Background*. Insomnia is a form of chronic sleep problem of public health importance which impacts the life of elderly people negatively. *Methods*. Cross-sectional study of 843 elderly patients aged 60 years and above who presented consecutively at Geriatric Centre, University College Hospital, Ibadan, Nigeria. The World Health Organization Composite International Diagnostic Interview was used to diagnose insomnia. We assessed the following candidate variables which may be associated with insomnia such as socidemographic characteristics, morbidities, and lifestyle habits. Statistical analysis was done with SPSS 17. *Results*. The point prevalence of insomnia was 27.5%. Insomnia was significantly associated with being female, not being currently married, having formal education, living below the poverty line, and not being physically active. Health complaints of abdominal pain, generalized body pain, and persistent headaches were significantly associated with insomnia. *Conclusion*. The high prevalence of insomnia among elderly patients in this setting calls for concerted effort by healthcare workers to educate the elderly on lifestyle modification.

## 1. Introduction

Chronic sleep problem is very common in elderly people [1]. Sufficient total sleep time as well as sleep that is in synchrony with the individual's circadian rhythm is required for a refreshing sleep [1]. More than half of elderly people have at least one chronic sleep problem [1]. In primary care settings, commonly encountered chronic sleep problems are insomnia and excessive daytime sleepiness [1, 2]. Insomnia is defined by the World Health Organization, using the Composite International Diagnostic Interview (CIDI) version 3, as any individual who has one of the following night-time sleep problems: difficulty in initiating sleep (DIS), difficulty in maintaining sleep (DMS), early morning awakening (EMA), and nonrestorative sleep (NRS) almost every night for ≥2 weeks [3].

The population of elderly people in Nigeria is increasing and it is expected to reach 15 million by the year 2025 [4]. There are changes in the sleep pattern as people age, with increasing prevalence of insomnia [1, 2, 5]. In Nigeria, the Ibadan Study of Aging (ISA) group reported an incidence of 8.0% and 25.7% for insomnia syndrome and insomnia symptoms, respectively, among the community-dwelling elderly people [3]. Elderly women were twice as likely as men to report difficulty falling asleep [6, 7]. Studies with clinical implications have added to the body of evidence that chronic sleep problems such as insomnia are not benign but rather an important risk marker for mortality in community-dwelling elderly [8]. In Nigeria, the ISA group reported a significant association between insomnia and chronic medical problems such as chronic pain and hypertension [9].

Community-based studies in Nigeria reported high prevalence of chronic sleep problems among the elderly [3, 9]. The paucity of data on the chronic sleep problems and the health implications has created a knowledge gap in its recognition and management by health workers. The main objective of this study was to assess the prevalence of insomnia and associated risk factors in elderly patients in a frontline ambulatory clinic.

## 2. Methods

### 2.1. Study Site

This study was carried out at the Chief Tony Anenih Geriatric Centre (CTAGC) of the University College Hospital (UCH), Ibadan. Ibadan is the capital city of Oyo State in the south-western area of Nigeria and has a population of 3.6 million inhabitants [10]. CTAGC is a purpose-built facility for the care of elderly people and the first in Nigeria. It was commissioned on 17 November 2012 and manages patients both on in- and outpatient basis. The centre has various speciality units such as physiotherapy, dietetics, geriatric lifestyle, ophthalmology, geriatric dentistry, memory, and geriatric psychiatry units. Elderly patients are comprehensively assessed using a checklist while those requiring further specialist care are referred to other specialty clinics within the University College Hospital, Ibadan.

### 2.2. Study Design

The cross-sectional design was used for this study.

### 2.3. Study Population

All consenting elderly patients (60 years and above) who presented during the period of the study (January 15 to April 30 2013) were recruited. Leslie Kish formula for single proportion was used to calculate the sample size using the best estimate of the prevalence of insomnia in elderly Nigerians [11] and 843 patients were recruited. Those who were too ill to participate in the study and those who did not consent were excluded.

### 2.4. Sampling Technique

Respondents were selected consecutively.

### 2.5. Procedure

The respondents were interviewed with a semistructured questionnaire which was pretested before use. The World Health Organization Composite International Diagnostic Interview version 3, a fully structured diagnostic interview (CIDI-3) asks questions about difficulty in initiating sleep (DIS), difficulty in maintaining sleep (DMS), early morning awakening (EMA), nonrestorative sleep (NRS), daytime sleepiness, and dissatisfaction with sleep [3]. The CIDI-3 asks questions about difficulty in initiating sleep (DIS), difficulty in maintaining sleep (DMS), early morning awakening (EMA), nonrestorative sleep (NRS), daytime sleepiness, and dissatisfaction with sleep. The CIDI questions have been adapted and used in a previous study on elderly in Ibadan, Nigeria [3]. Insomnia was assigned to respondent who endorsed any one of the four night-time sleep problems (DIS, DMS, EMA, or NRS) almost every night for ≥2 weeks [3].

Detailed history and comprehensive physical examination of the respondents were carried out by the researchers who are physicians. The questionnaire was translated into Yoruba (the local dialect of most respondents) and independently back-translated to English language. It was then field-tested to ensure that the original meaning was retained. The questionnaire took about 40 minutes to be administered.

### 2.6. Anthropometric Measurements

Height was recorded to the nearest centimetre with a measurement stand (stadiometer) which was positioned on a flat surface. The respondents were asked to remove their shoes, and their heels were positioned against the stand with their scapula, buttocks, and heels resting against the wall. Weight was recorded to the nearest 0.1 kg. Respondents stood on the weighing scale which was placed on a flat horizontal surface, after removal of their personal effects. The readings were made by the researcher standing in front of the respondents and the zero mark was checked after every reading for accuracy.

The BMI of the patients was calculated by dividing weight (kilogrammes) by height in meters squared and this was graded using the WHO anthropometric classification [12]. Underweight was defined as BMI < 18.4 kg/m^2^ and 18.5–24.9 kg/m^2^ was defined as normal. Overweight was BMI 25.0–29.9 kg/m^2^; class I obesity was defined as BMI 30.0 to 34.9 kg/m^2^, class II obesity was defined as BMI 35.0–39.9 kg/m^2^, and Class III obesity, which is morbid obesity, was defined as BMI of greater than 40.0 kg/m^2^ [12].

### 2.7. Waist-Hip Ratio (WHR)

The waist and hip circumferences were measured using a flexible nonelastic measuring tape and these were measured to the nearest 0.1 cm. The hip circumference was measured at a level parallel to the floor, at the largest circumference of the buttocks. The waist circumference was measured at the end of several consecutive natural breaths, at a level parallel to the floor, midpoint between the top of the iliac crest and the lower margin of the last palpable rib in the mid axillary line. The waist circumference was used to identify individuals with increased risks for metabolic complications based upon threshold values of 80 cm or greater for women and 94 cm or greater for men as defined by the World Health Organization (WHO) and International Diabetic Federation (IDF) [13]. Waist to Hip ratio (WHR) was estimated by dividing waist circumference by hip circumference. The WHR threshold used for elderly women was 0.85 or more and for men was 1.00 or more [13, 14].

### 2.8. Neck Circumference

It was measured with a flexible inelastic measuring tape and recorded to the nearest 0.1 cm. Neck circumferences greater than 40 cm in women and 43 cm in men correlate strongly with the development of obstructive sleep apnea and have been adopted as the upper limit for both genders [15].

### 2.9. Throat Examination

Oropharyngeal crowding was assessed during throat examination using the Mallampati visual assessment classification (see Appendix/Figure 2) [16]. This was classified as follows: class I: tonsils, pillars, and soft palate were clearly visible; class II: the uvula, pillars, and upper pole were visible; class III: only part of the soft palate was visible; the tonsils, pillars, and base of the uvula could not be seen; and class IV: only the hard palate was visible [16].

## 3. Ethical Consideration

### 3.1. Consent for the Study

Ethical approval was received from the University of Ibadan/UCH Institutional Ethical Review Board (NHREC/05/01/2008a). Informed consent of each respondent was obtained before examination and administration of questionnaire.

### 3.2. Respondent's Follow-Up

All the elderly patients recruited were given health education and counselling on their health complaints. They were treated for their primary complaints and those needing further evaluation were referred to other specialist units within the hospital facility for further management of their conditions.

### 3.3. Data Analysis

At the end of each day of the study, the administered questionnaires were sorted out, cross-checked after each interview, and coded serially. Data entering, cleaning, and analysis were carried out using SSPS (version 17). Descriptive statistics was used to describe sociodemographic characteristics of the respondents. Appropriate charts were used to illustrate categorical variables. Chi-square statistics was used to assess association between categorical variables and Student's *t*-test to test association between continuous variables. The values of significance were set at *P* ≤ 0.05. Logistic regression analysis was used to explore relationship between significant variables and insomnia.

## 4. Results

There were 340 (40.3%) male and 503 (59.7%) female respondents. Their mean (SD) age was 69.3 (7.1) with a range of 60–98 years. The modal age group of the males was 65–69 years, while for the females it was 60–64 years. Majority (86.2%) of the men were currently married while half (52.5%) of the women were widowed. The greatest proportion (50.1%) of the women had no formal education, while the highest proportion (27.6%) of the men attained tertiary education. Half (52.1%) of the male respondents had six or more children, while a higher proportion (62.4%) of the female respondents had less than six children; see Table 1.

The point prevalence of insomnia was 27.5%. The prevalence of insomnia was significantly higher among the women compared with the men (30.2% versus 23.5%, *χ* ^2^ = 4.551; *P* = 0.033). Respondents who were not currently married (31.7%) had significant higher prevalence of insomnia compared with those who were currently married (24.9%) (*χ* ^2^ = 4.718, *P* = 0.019). Significantly, higher proportion of respondents with formal education (32.8%) had insomnia compared with those who had no formal education (24.1%) (*χ* ^2^ = 7.744, *P* = 0.004). Respondents who were currently engaged in occupational activities had higher prevalence of insomnia compared with those not currently engaged in occupational activities (30.2% versus 26.5%) without a statistically significant difference (*χ* ^2^ = 1.128, *P* = 0.164). Respondents who were living (28.6%) and depended financially (28.0%) on others such as spouse, children, grandchildren, and relatives had higher prevalence of insomnia compared with those who were self-supporting (25.8%) and who lived alone (19.8%). The prevalence of insomnia was significantly higher among respondents living below the World Bank defined poverty line of $1.25 per day compared with those living above the poverty line (34.7% versus 23.6%) (*χ* ^2^ = 11.783, *P* < 0.0001); see Table 2.

The lifestyle habits and hospital care utilization pattern of the respondents were shown in Table 3. Insomnia was more common without statistical difference among respondents who drank alcohol (34.0% versus 27.1%), smoked tobacco (37.5% versus 27.3%), took cannabis (50.0% versus 27.2%), drank coffee (32.3% versus 27.5%), and were not engaged in physical activities (34.8% versus 26.4%) compared with those who did not engage in these lifestyle habits. The proportion of respondents with insomnia decreased significantly with the increased level of reported physical activities from those who were active (35.4%) through those who were moderately active (27.8%) to those who were very active (22.6%) (*χ* ^2^ = 6.062; *P* = 0.048). Hospital care utilization pattern showed insomnia to be more common without statistical difference among respondents who visited hospital four or more times (28.1% versus 26.8%), were previously hospitalized (30.1% versus 25.9%), and were first hospitalized after the age of 60 years (32.0% versus 26.4%) compared with those who visited the hospital less than four times, never got hospitalized, and were hospitalized before the age of 60 years.

**Figure 1:**
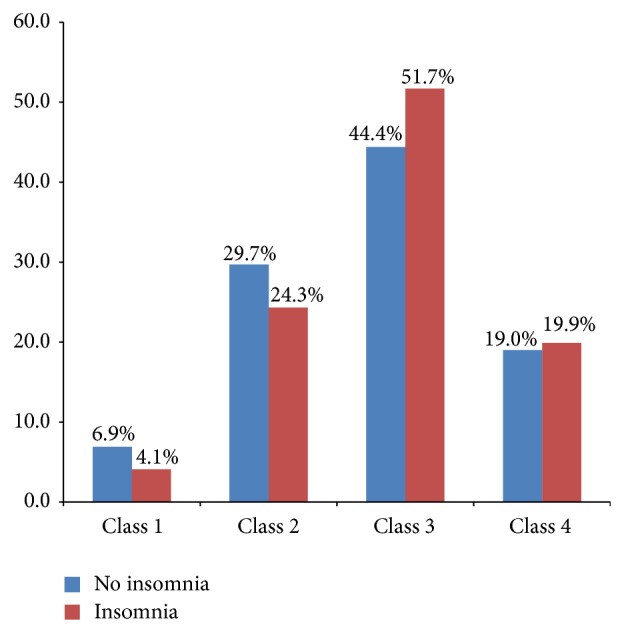
Oropharyngeal crowding (Mallampati classification) by prevalence of insomnia.

**Figure 2:**
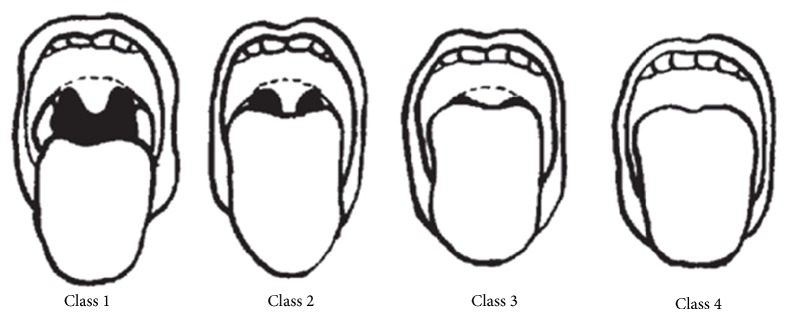
Oropharyngeal crowding (Mallampati classification).

The prevalence of insomnia was significantly associated with the complaints of abdominal discomfort (OR = 1.83, *P* = 0.032), generalized body pain (OR = 1.72, *P* = 0.001), and persistent headaches (OR = 1.93, *P* = 0.040), see Table 4. The oropharyngeal crowding in the respondents using the Mallampati classification is shown in Figure 1. Mallampati classes 1 and 2 had higher proportions of respondents who had no insomnia compared with those diagnosed with insomnia. Conversely, higher proportions of respondents with insomnia were in classes 3 and 4 when compared with those without insomnia.

The mean time estimated by the respondents to fall asleep was significantly higher in those with insomnia (25.9 ± 9.4 minutes) compared with those without insomnia (12.2 ± 2.4 minutes) (*t* = 10.023; *P* < 0.0001). Significantly, respondents without insomnia had more hours of sleep during the night compared with those with insomnia (6.9 ± 1.6 hours versus 4.7 ± 1.7 hours, *t* = 43.316; *P* < 0.0001). When asked to estimate the total hours of sleep in a day (24 hours), respondents without insomnia enjoyed more total hours of sleep compared with those with insomnia (9.2 ± 2.0 hours versus 9.0 ± 2.2 hours, *t* = 60.642; *P* < 0.0001).

Table 5 shows the anthropometric measurements by the prevalence of insomnia. Among the males, respondents with waist-hip ratio (WHR) of ≥0.90 had higher prevalence of insomnia when compared with those with WHR of <0.90 without significant difference (25.2% versus 13.0%) (*χ* ^2^ = 3.251; *P* = 0.071). Among the females, respondents with neck circumference of ≥40 cm had higher prevalence of insomnia when compared with those with neck circumference of <40 cm without significant difference (31.6% versus 30.2%) (*χ* ^2^ = 0.017; *P* = 0.895). None of the variables found significant in the bivariate analysis remain so in the final multivariate model.

## 5. Discussion

This hospital-based study was carried out among 843 elderly respondents with a female preponderance. This was comparable to the Ibadan study on ageing by Gureje et al. in 2011 in which there was a higher proportion of female respondents though their study was community based [3]. Globally, life expectancy is more favourable for women than men, 65.9 years for women as compared with 59.4 years for men [17]. We used 60 years as the cut-off for the elderly in this study because of the low life expectancy in the developing countries especially Nigeria which was 51 and 52 years for males and females, respectively [18]. Similarly, the United Nations designated the elderly as people aged 60 years and above [19].

In this study, the point prevalence of insomnia was found to be 27.5%. This was similar to one prospective cohort study in United State of America (USA) that found 23 to 34% of elderly people with insomnia [5]. Similarly, a Chinese study reported a prevalence of chronic insomnia of 4–22% [15]. A study in Nigeria among community-dwelling elderly people indicated an incidence of 25.7% for insomnia symptoms [3].

Female respondents had a significant higher prevalence of insomnia than men. In general, insomnia symptoms are more prevalent in women than in men and tended to increase with age [20]. This was corroborated by the Ibadan study of ageing in Nigeria [3]. Widowhood, depression and vulnerability to chronic physical conditions have been reported in older women [3, 21]. Our study found no association between age and insomnia. This finding is similar to a community-based study in the same location where this study was conducted but was dissimilar to the report of a survey in the USA which reported that the prevalence of insomnia increased with age [3, 5].

Marital status was found to be significantly associated with insomnia in our study as about a third of those who were not currently married had insomnia compared with a quarter of those who were currently married. This was in contrast to Gureje et al. who reported higher levels of insomnia in the married respondents [3]. Those who were classified as not currently married in our study included the widows and those who were either separated or divorced from their spouses. Studies have shown a relationship between widowhood and insomnia [3, 21].

Occupation played an important role in the prevalence of insomnia as respondents who were employed had a higher prevalence of insomnia. This was corroborated by Abamara in 2012 among low cadre workers in South Eastern Nigeria in which he found that low cadre workers had more insomnia than others. The female workers attributed their insomnia to the domestic chores normally done after closing hours, with the males resorting to drinking alcohol such as beer and smoking cigarettes or Indian hemp which may initiate insomnia [22].

We employed the World Bank definition of the abject poverty and found a strong association between insomnia and living below the poverty line of less than $1.25 per day. The Centre for Disease Control and Prevention (CDC) of USA reported higher prevalence of insomnia among adults living below the poverty level (24.8%) compared with those living above the poverty level (15.8%) [23]. Poverty is most likely to be found among low cadre worker who often engage in poor lifestyle habits such as excessive drinking of alcohol and tobacco and cannabis smoking [22].

Insomnia was found to be more common among respondents who consumed alcohol, smoked tobacco, took cannabis, and consumed coffee. This was corroborated by the study which found that substance abuse such as the use of cigarettes, Indian hemp, and alcohol contributed significantly to the causes of insomnia [22]. Those who lived a sedentary lifestyle were found to have more insomnia in our study as the prevalence of insomnia was inversely associated with the level of physical activities. Excessive daytime sleepiness following insomnia has been found to be strongly associated with reduced physical activities [24].

In elderly people, the problem of comorbidity in insomnia is important [25]. Insomnia is associated with poor health, depression, angina, limitations in activities of daily living, and use of benzodiazepines [7]. Across different populations, several studies have found a significant deleterious effect of sleep disturbances on self-rated health, incidence of cardio-metabolic diseases, quality of life, and mortality [3, 7, 15]. In our study, the prevalence of insomnia was higher in those respondents who had abdominal discomfort, generalized body pain, and continuous headaches. Hospital care utilization pattern demonstrated that insomnia was more among respondents who visited hospital over four times, had been hospitalized in the past, and had their first hospitalization over age of 60 years.

Most studies reported a direct association between insomnia and obesity [24, 26, 27]. But our study found no significant association between the prevalence of insomnia and generalized obesity (BMI) and central obesity (WHR) measures. This could be due to racial and cultural differences in the perception of insomnia and/or the presence of chronic morbidities like diabetes mellitus, hay fever, arthritis, and depression [13, 24].

Oropharyngeal crowding among respondents was graded using the Mallampati classification. Insomnia was more prevalent among respondents in classes 3 and 4. High Mallampati score has been found to be strongly associated with obstructive sleep apnoea which in turn leads to poor sleep [16]. The relative risk of individuals in the Mallampati class 3 or 4 having obstructive sleep apnoea was estimated to be twice those in Mallampati class 1 or 2 [16].

## 6. Conclusion

The proportion of elderly patients with insomnia in our setting is high, and in view of the increasing population of the elderly in Nigeria, as well as associated clinical morbidities, it is important to evaluate insomnia during routine clinic consultations. More research on sleep disorders such as insomnia needs to be done among the elderly who are more prone to cardiovascular and other comorbid medical conditions [28].

## Figures and Tables

**Table 1:** Sociodemographic characteristics.

	Male = 340 *n* (%)	Females = 503 *n* (%)	Total = 843 *N* (%)
Age groups (years)			
60–64	83 (24.4)	151 (30.0)	234 (27.8)
65–69	98 (28.8)	123 (24.5)	221 (26.2)
70–74	72 (21.2)	119 (23.7)	191 (22.7)
75–79	48 (14.1)	51 (10.1)	99 (11.7)
≥80	39 (11.5)	59 (11.7)	98 (11.6)
Marital status			
Married	293 (86.2)	222 (44.1)	515 (61.1)
Widowed	38 (11.2)	264 (52.5)	302 (35.8)
Separated	5 (1.5)	8 (1.6)	13 (1.5)
Divorced	3 (0.9)	8 (1.6)	11 (1.3)
Single	1 (0.3)	1 (0.2)	2 (0.2)
Formal education			
None	80 (23.5)	252 (50.1)	332 (39.4)
Primary	80 (23.5)	100 (19.9)	180 (21.4)
Secondary	86 (25.3)	70 (13.9)	156 (18.5)
Tertiary	94 (27.6)	81 (16.1)	175 (20.8)
Occupational activities			
Not currently engaged in occupational activities	225 (75.0)	356 (70.8)	611 (72.5)
Currently engaged in occupational activities	85 (25.0)	147 (29.2)	232 (27.5)
Living arrangement			
Alone	41 (12.7)	65 (13.5)	106 (13.2)
With spouse	247 (76.2)	197 (41.0)	444 (55.2)
With children/grandchildren	32 (9.9)	197 (41.0)	229 (28.4)
With relatives/friends	4 (1.2)	22 (4.6)	26 (3.2)
Financial support			
Self	109 (32.3)	85 (17.1)	194 (23.2)
Spouse	8 (2.4)	13 (2.6)	21 (2.5)
Children/grandchildren	210 (62.3)	390 (78.5)	600 (71.9)
Relatives/friends	10 (3.0)	9 (1.8)	19 (2.3)
Number of children			
0–5	163 (47.9)	314 (62.4)	477 (56.6)
≥6	177 (52.1)	189 (37.6)	366 (43.4)
Income			
Below the poverty line (<$1.25 per day)	89 (26.2)	208 (41.4)	297 (35.2)
Above the poverty line (≥$1.25 per day)	251 (73.8)	295 (58.6)	546 (64.8)

**Table 2:** Sociodemographic characteristics and the prevalence of insomnia.

	Insomnia		
	Yes = 232 *n* (%)	No = 611 *n* (%)	
Age groups (years)			
60–64	70 (29.9)	164 (70.1)	*χ* ^2^ = 4.123 *P* = 0.390
65–69	66 (29.9)	155 (70.1)	
70–74	47 (24.6)	144 (75.4)	
75–79	21 (21.2)	78 (78.8)	
≥80	28 (28.6)	70 (71.4)	
Sex			
Males	80 (23.5)	260 (76.5)	*χ* ^2^ = 4.551 *P* = 0.033^*^
Females	152 (30.2)	351 (69.8)	
Marital status			
Currently married	128 (24.9)	387 (75.1)	*χ* ^2^ = 4.718 *P* = 0.019^*^
Not currently married	104 (31.7)	224 (68.3)	
Education			
None	123 (24.1)	388 (75.9)	*χ* ^2^ = 7.744 *P* = 0.004^*^
Had formal education	109 (32.8)	223 (67.2)	
Occupational activities			
Not currently engaged in occupational activities	162 (26.5)	449 (73.5)	*χ* ^2^ = 1.128 *P* = 0.164
Currently engaged in occupational activities	70 (30.2)	162 (69.8)	
Living arrangement			
Alone	21 (19.8)	85 (80.2)	*χ* ^2^ = 3.613 *P* = 0.057
With others	211 (28.6)	526 (71.4)	
Financial support			
Self	50 (25.8)	144 (74.2)	*χ* ^2^ = 0.386 *P* = 0.535
Others	182 (28.0)	467 (72.0)	
Number of children			
0–5	127 (26.6)	350 (73.4)	*χ* ^2^ = 0.442 *P* = 0.506
≥6	105 (28.7)	261 (71.3)	
Income			
Below the poverty line (<$1.25 per day)	103 (34.7)	194 (65.3)	*χ* ^2^ = 11.783 *P* < 0.0001^*^
Above the poverty line (≥$1.25 per day)	129 (23.6)	417 (76.4)	

^*^Significant at 5% level of significance.

**Table 3:** Lifestyle habits and hospital care utilization by the prevalence of insomnia.

	Insomnia		Total = 843 *N* (%)
	Yes = 232 *n* (%)	No = 611 *n* (%)	
Alcohol			
Yes	18 (34.0)	35 (66.0)	53 (100.0)
No	214 (27.1)	576 (72.9)	790 (100.0)
*χ* ^2^ = 1.176, df = 1, *P* = 0.278			
Tobacco			
Yes	6 (37.5)	10 (62.5)	16 (100.0)
No	226 (27.3)	601 (72.7)	827 (100.0)
*χ* ^2^ = 0.814, df = 1, *P* = 0.367^†^			
Cannabis			
Yes	4 (50.0)	4 (50.0)	8 (100.0)
No	228 (27.2)	607 (72.8)	835 (100.0)
*χ* ^2^ = 2.046, df = 1, *P* = 0.153^†^			
Coffee			
Yes	10 (32.3)	21 (67.7)	31 (100.0)
No	222 (27.5)	590 (72.5)	812 (100.0)
*χ* ^2^ = 0.362, df = 1, *P* = 0.547			
Engagement in physical activities			
Yes	193 (26.4)	538 (73.6)	731 (100.0)
No	39 (34.8)	73 (65.2)	112 (100.0)
*χ* ^2^ = 3.451, df = 1, *P* = 0.632			
Level of physical activities			
Not active	40 (35.4)	73 (64.6)	113 (100.0)
Moderately active	145 (27.8)	377 (72.2)	522 (100.0)
Very active	47 (22.6)	161 (77.4)	208 (100.0)
*χ* ^2^ = 6.062, df = 2, *P* = 0.048^*^			
Hospital visits in the past 12 months			
0–3 times	95 (26.8)	260 (73.2)	355 (100.0)
≥4 times	137 (28.1)	351 (71.9)	488 (100.0)
*χ* ^2^ = 0.178, df = 1, *P* = 0.673			
Previous hospital admission			
Yes	99 (30.1)	230 (69.9)	329 (100.0)
No	133 (25.9)	381 (74.1)	514 (100.0)
*χ* ^2^ = 1.787, df = 1, *P* = 0.181			
Age at first hospital admission			
Never or before the age of 60 years	178 (26.4)	496 (73.4)	674 (100.0)
After the age of 60 years	54 (32.0)	115 (68.0)	169 (100.0)
*χ* ^2^ = 2.081, df = 1, *P* = 0.149			
Has been on regular medications in the past one month			
Yes	106 (27.2)	276 (72.3)	382 (100.0)
No	126 (27.3)	335 (72.7)	461 (100.0)
*χ* ^2^ = 3.451, df = 1, *P* = 0.063			

^†^Yates corrected, ^*^Significant at 5% level of significance.

**Table 4:** Morbidities by prevalence of insomnia.

Morbidities	Insomnia		Odds ratio	95% CI	*P*
	Yes = 232 *n* (%)	No = 611 *n* (%)			
Abdominal discomfort	22 (40.0)	33 (60.0)	1.83	1.05–3.20	0.032^*^
Generalized body pain	77 (36.0)	137 (64.0)	1.72	1.23–2.40	0.001^*^
Breathlessness	7 (33.3)	14 (66.7)	1.33	0.54–3.26	0.546
Chest pain	4 (25.0)	12 (75.0)	0.88	0.29–2.65	0.374
Fever	2 (16.7)	10 (83.3)	0.52	0.12–2.25	0.528
Severe cough	10 (35.7)	18 (64.3)	1.48	0.68–3.22	0.324
Psychosomatic symptoms	10 (40.0)	15 (60.0)	1.79	0.81–3.98	0.156
Diabetes mellitus	17 (24.6)	52 (75.4)	0.85	0.48–1.50	0.531
Lower urinary tract symptoms	11 (34.4)	21 (65.6)	1.40	0.67–2.91	0.376
Generalized body weakness	5 (27.8)	13 (72.2)	1.01	0.37–2.79	0.953
Persistent headaches	17 (41.5)	24 (58.5)	1.93	1.03–3.64	0.040^*^
Hypertension	49 (25.9)	140 (74.1)	0.90	0.62–1.30	0.577

^*^Significant at 5% level of significance.

**Table 5:** Anthropometric measurements by prevalence of insomnia.

	Insomnia		
	Yes = 232 *n* (%)	No = 611 *n* (%)	Total = 843 *N* (%)
Waist circumference			
** **Males			
** **<94 cm	47 (25.7)	136 (74.6)	183 (100.0)
** **≥94 cm	33 (21.0)	124 (79.0)	157 (100.0)
** ** *χ* ^2^ = 1.022, df = 1, *P* = 0.312			
** **Females			
** **<80 cm	15 (32.6)	31 (67.4)	46 (100.0)
** **≥80 cm	137 (30.0)	320 (70.0)	457 (100.0)
** ** *χ* ^2^ = 0.137, df = 1, *P* = 0.711			
Waist-hip ratio (WHR)			
** **Males			
** **<0.90	6 (13.0)	40 (87.0)	46 (100.0)
** **≥0.90	74 (25.2)	220 (74.8)	294 (100.0)
** ** *χ* ^2^ = 3.251, df = 1, *P* = 0.071			
** **Females			
** **<0.85	15 (32.6)	31 (67.4)	46 (100.0)
** **≥0.85	137 (30.0)	320 (70.0)	457 (100.0)
** ** *χ* ^2^ = 0.374, df = 1, *P* = 0.541			
Neck circumference			
** **Males			
** **<43 cm	75 (23.9)	239 (76.1)	314 (100.0)
** **≥43 cm	5 (19.2)	21 (80.8)	26 (100.0)
** ** *χ* ^2^ = 0.289, df = 1, *P* = 0.591			
** **Females			
** **<40 cm	146 (30.2)	338 (69.8)	484 (100.0)
** **≥40 cm	6 (31.6)	13 (68.4)	19 (100.0)
** ** *χ* ^2^ = 0.017, df = 1, *P* = 0.895			
Body mass index (BMI)			
** **Males			
** **Not obese (<30 kg/m^2^)	72 (24.7)	220 (75.3)	292 (100.0)
** **Obese (≥30 kg/m^2^)	8 (16.7)	40 (83.3)	48 (100.0)
** ** *χ* ^2^ = 1.463, df = 1, *P* = 0.226			
** **Females			
** **Not obese (<30 kg/m^2^)	111 (33.0)	225 (67.0)	336 (100.0)
** **Obese (≥30 kg/m^2^)	41 (24.6)	126 (75.4)	167 (100.0)
** ** *χ* ^2^ = 3.809, df = 1, *P* = 0.051			

## Appendix

See Figure 2.
